# How can resilience moderate the effects of the COVID-19 pandemic on mental health?

**DOI:** 10.1192/j.eurpsy.2022.502

**Published:** 2022-09-01

**Authors:** C. Ciampi, M. Carfagno, M. Raia, V. Giallonardo, V. Del Vecchio, M. Luciano, G. Sampogna, A. Fiorillo

**Affiliations:** University of Campania “Luigi Vanvitelli”, Department Of Psychiatry, Naples, Italy

**Keywords:** Depression, coping strategies, Covid-19, resilience

## Abstract

**Introduction:**

The COVID-19 pandemic represents a new form of trauma, which is impacting on the mental health of the general population. However, the effects of this new trauma are variable, being mediated by individual factors such as the levels of resilience and the coping strategies.

**Objectives:**

The aims of the present study are: 1) describe the levels of resilience and the type of coping strategies adopted by the Italian general adult population during the first wave of the pandemic; 2) evaluate the protective role of coping strategies and resilience on the levels of depressive, anxiety and stress symptoms.

**Methods:**

An online survey has been developed, which includes several validated self-reported questionnaires for the evaluation of participants’ mental health condition, coping strategies and levels of resilience. The main outcome measure is the Depression Anxiety and Stress Scale-21 (DASS-21).

**Results:**

The finale sample consists of 20,720 participants, more than half reported low levels of resilience, which were not associated with age or gender. The levels of resilience did not differ among the general population, patients with pre-existing mental disorders and those infected by COVID-19. People with low levels of resilience rarely used adaptive coping strategies. The levels of resilience did not have any influence on stress, depressive or anxiety symptoms.

**Conclusions:**

The presence of low levels of resilience in the general population may be the missing link between the pandemic and increasing concerns on mental health problems. This could be important for the development of ad-hoc supportive and preventive psychosocial interventions.

**Disclosure:**

No significant relationships.

